# The use of Big Data Analytics in healthcare

**DOI:** 10.1186/s40537-021-00553-4

**Published:** 2022-01-06

**Authors:** Kornelia Batko, Andrzej Ślęzak

**Affiliations:** 1 Department of Business Informatics, University of Economics in Katowice, Katowice, Poland; 2 Department of Biomedical Processes and Systems, Institute of Health and Nutrition Sciences, Częstochowa University of Technology, Częstochowa, Poland

**Keywords:** Big Data, Big Data Analytics, Data-driven healthcare

## Abstract

The introduction of Big Data Analytics (BDA) in healthcare will allow to use new technologies both in treatment of patients and health management. The paper aims at analyzing the possibilities of using Big Data Analytics in healthcare. The research is based on a critical analysis of the literature, as well as the presentation of selected results of direct research on the use of Big Data Analytics in medical facilities. The direct research was carried out based on research questionnaire and conducted on a sample of 217 medical facilities in Poland. Literature studies have shown that the use of Big Data Analytics can bring many benefits to medical facilities, while direct research has shown that medical facilities in Poland are moving towards data-based healthcare because they use structured and unstructured data, reach for analytics in the administrative, business and clinical area. The research positively confirmed that medical facilities are working on both structural data and unstructured data. The following kinds and sources of data can be distinguished: from databases, transaction data, unstructured content of emails and documents, data from devices and sensors. However, the use of data from social media is lower as in their activity they reach for analytics, not only in the administrative and business but also in the clinical area. It clearly shows that the decisions made in medical facilities are highly data-driven. The results of the study confirm what has been analyzed in the literature that medical facilities are moving towards data-based healthcare, together with its benefits.

## Introduction

The main contribution of this paper is to present an analytical overview of using structured and unstructured data (Big Data) analytics in medical facilities in Poland. Medical facilities use both structured and unstructured data in their practice. Structured data has a predetermined schema, it is extensive, freeform, and comes in variety of forms [27]. In contrast, unstructured data, referred to as Big Data (BD), does not fit into the typical data processing format. Big Data is a massive amount of data sets that cannot be stored, processed, or analyzed using traditional tools. It remains stored but not analyzed. Due to the lack of a well-defined schema, it is difficult to search and analyze such data and, therefore, it requires a specific technology and method to transform it into value [20, 68]. Integrating data stored in both structured and unstructured formats can add significant value to an organization [27]. Organizations must approach unstructured data in a different way. Therefore, the potential is seen in Big Data Analytics (BDA). Big Data Analytics are techniques and tools used to analyze and extract information from Big Data. The results of Big Data analysis can be used to predict the future. They also help in creating trends about the past. When it comes to healthcare, it allows to analyze large datasets from thousands of patients, identifying clusters and correlation between datasets, as well as developing predictive models using data mining techniques [60].

This paper is the first study to consolidate and characterize the use of Big Data from different perspectives. The first part consists of a brief literature review of studies on Big Data (BD) and Big Data Analytics (BDA), while the second part presents results of direct research aimed at diagnosing the use of big data analyses in medical facilities in Poland.

Healthcare is a complex system with varied stakeholders: patients, doctors, hospitals, pharmaceutical companies and healthcare decision-makers. This sector is also limited by strict rules and regulations. However, worldwide one may observe a departure from the traditional doctor-patient approach. The doctor becomes a partner and the patient is involved in the therapeutic process [14]. Healthcare is no longer focused solely on the treatment of patients. The priority for decision-makers should be to promote proper health attitudes and prevent diseases that can be avoided [81]. This became visible and important especially during the Covid-19 pandemic [44].

The next challenges that healthcare will have to face is the growing number of elderly people and a decline in fertility. Fertility rates in the country are found below the reproductive minimum necessary to keep the population stable [10]. The reflection of both effects, namely the increase in age and lower fertility rates, are demographic load indicators, which is constantly growing. Forecasts show that providing healthcare in the form it is provided today will become impossible in the next 20 years [70]. It is especially visible now during the Covid-19 pandemic when healthcare faced quite a challenge related to the analysis of huge data amounts and the need to identify trends and predict the spread of the coronavirus. The pandemic showed it even more that patients should have access to information about their health condition, the possibility of digital analysis of this data and access to reliable medical support online. Health monitoring and cooperation with doctors in order to prevent diseases can actually revolutionize the healthcare system. One of the most important aspects of the change necessary in healthcare is putting the patient in the center of the system.

Technology is not enough to achieve these goals. Therefore, changes should be made not only at the technological level but also in the management and design of complete healthcare processes and what is more, they should affect the business models of service providers. The use of Big Data Analytics is becoming more and more common in enterprises [17, 54]. However, medical enterprises still cannot keep up with the information needs of patients, clinicians, administrators and the creator’s policy. The adoption of a Big Data approach would allow the implementation of personalized and precise medicine based on personalized information, delivered in real time and tailored to individual patients.

To achieve this goal, it is necessary to implement systems that will be able to learn quickly about the data generated by people within clinical care and everyday life. This will enable data-driven decision making, receiving better personalized predictions about prognosis and responses to treatments; a deeper understanding of the complex factors and their interactions that influence health at the patient level, the health system and society, enhanced approaches to detecting safety problems with drugs and devices, as well as more effective methods of comparing prevention, diagnostic, and treatment options [40].

In the literature, there is a lot of research showing what opportunities can be offered to companies by big data analysis and what data can be analyzed. However, there are few studies showing how data analysis in the area of healthcare is performed, what data is used by medical facilities and what analyses and in which areas they carry out. This paper aims to fill this gap by presenting the results of research carried out in medical facilities in Poland. The goal is to analyze the possibilities of using Big Data Analytics in healthcare, especially in Polish conditions. In particular, the paper is aimed at determining what data is processed by medical facilities in Poland, what analyses they perform and in what areas, and how they assess their analytical maturity. In order to achieve this goal, a critical analysis of the literature was performed, and the direct research was based on a research questionnaire conducted on a sample of 217 medical facilities in Poland. It was hypothesized that medical facilities in Poland are working on both structured and unstructured data and moving towards data-based healthcare and its benefits. Examining the maturity of healthcare facilities in the use of Big Data and Big Data Analytics is crucial in determining the potential future benefits that the healthcare sector can gain from Big Data Analytics. There is also a pressing need to predicate whether, in the coming years, healthcare will be able to cope with the threats and challenges it faces.

This paper is divided into eight parts. The first is the introduction which provides background and the general problem statement of this research. In the second part, this paper discusses considerations on use of Big Data and Big Data Analytics in Healthcare, and then, in the third part, it moves on to challenges and potential benefits of using Big Data Analytics in healthcare. The next part involves the explanation of the proposed method. The result of direct research and discussion are presented in the fifth part, while the following part of the paper is the conclusion. The seventh part of the paper presents practical implications. The final section of the paper provides limitations and directions for future research.

## Considerations on use Big Data and Big Data Analytics in the healthcare

In recent years one can observe a constantly increasing demand for solutions offering effective analytical tools. This trend is also noticeable in the analysis of large volumes of data (Big Data, BD). Organizations are looking for ways to use the power of Big Data to improve their decision making, competitive advantage or business performance [7, 54]. Big Data is considered to offer potential solutions to public and private organizations, however, still not much is known about the outcome of the practical use of Big Data in different types of organizations [24].

As already mentioned, in recent years, healthcare management worldwide has been changed from a disease-centered model to a patient-centered model, even in value-based healthcare delivery model [68]. In order to meet the requirements of this model and provide effective patient-centered care, it is necessary to manage and analyze healthcare Big Data.

The issue often raised when it comes to the use of data in healthcare is the appropriate use of Big Data. Healthcare has always generated huge amounts of data and nowadays, the introduction of electronic medical records, as well as the huge amount of data sent by various types of sensors or generated by patients in social media causes data streams to constantly grow. Also, the medical industry generates significant amounts of data, including clinical records, medical images, genomic data and health behaviors. Proper use of the data will allow healthcare organizations to support clinical decision-making, disease surveillance, and public health management. The challenge posed by clinical data processing involves not only the quantity of data but also the difficulty in processing it.

In the literature one can find many different definitions of Big Data. This concept has evolved in recent years, however, it is still not clearly understood. Nevertheless, despite the range and differences in definitions, Big Data can be treated as a: large amount of digital data, large data sets, tool, technology or phenomenon (cultural or technological.

Big Data can be considered as massive and continually generated digital datasets that are produced via interactions with online technologies [53]. Big Data can be defined as datasets that are of such large sizes that they pose challenges in traditional storage and analysis techniques [28]. A similar opinion about Big Data was presented by Ohlhorst who sees Big Data as extremely large data sets, possible neither to manage nor to analyze with traditional data processing tools [57]. In his opinion, the bigger the data set, the more difficult it is to gain any value from it.

In turn, Knapp perceived Big Data as tools, processes and procedures that allow an organization to create, manipulate and manage very large data sets and storage facilities [38]. From this point of view, Big Data is identified as a tool to gather information from different databases and processes, allowing users to manage large amounts of data.

Similar perception of the term ‘Big Data’ is shown by Carter. According to him, Big Data technologies refer to a new generation of technologies and architectures, designed to economically extract value from very large volumes of a wide variety of data by enabling high velocity capture, discovery and/or analysis [13].

Jordan combines these two approaches by identifying Big Data as a complex system, as it needs data bases for data to be stored in, programs and tools to be managed, as well as expertise and personnel able to retrieve useful information and visualization to be understood [37].

Following the definition of Laney for Big Data, it can be state that: it is large amount of data generated in very fast motion and it contains a lot of content [43]. Such data comes from unstructured sources, such as stream of clicks on the web, social networks (Twitter, blogs, Facebook), video recordings from the shops, recording of calls in a call center, real time information from various kinds of sensors, RFID, GPS devices, mobile phones and other devices that identify and monitor something [8]. Big Data is a powerful digital data silo, raw, collected with all sorts of sources, unstructured and difficult, or even impossible, to analyze using conventional techniques used so far to relational databases.

While describing Big Data, it cannot be overlooked that the term refers more to a phenomenon than to specific technology. Therefore, instead of defining this phenomenon, trying to describe them, more authors are describing Big Data by giving them characteristics included a collection of V’s related to its nature [2, 3, 23, 25, 58]:

- Volume (refers to the amount of data and is one of the biggest challenges in Big Data Analytics),
- Velocity (speed with which new data is generated, the challenge is to be able to manage data effectively and in real time),
- Variety (heterogeneity of data, many different types of healthcare data, the challenge is to derive insights by looking at all available heterogenous data in a holistic manner),
- Variability (inconsistency of data, the challenge is to correct the interpretation of data that can vary significantly depending on the context),
- Veracity (how trustworthy the data is, quality of the data),
- Visualization (ability to interpret data and resulting insights, challenging for Big Data due to its other features as described above).
- Value (the goal of Big Data Analytics is to discover the hidden knowledge from huge amounts of data).

Big Data is defined as an information asset with high volume, velocity, and variety, which requires specific technology and method for its transformation into value [21, 77]. Big Data is also a collection of information about high-volume, high volatility or high diversity, requiring new forms of processing in order to support decision-making, discovering new phenomena and process optimization [5, 7]. Big Data is too large for traditional data-processing systems and software tools to capture, store, manage and analyze, therefore it requires new technologies [28, 50, 61] to manage (capture, aggregate, process) its volume, velocity and variety [9].

Undoubtedly, Big Data differs from the data sources used so far by organizations. Therefore, organizations must approach this type of unstructured data in a different way. First of all, organizations must start to see data as flows and not stocks—this entails the need to implement the so-called streaming analytics [48]. The mentioned features make it necessary to use new IT tools that allow the fullest use of new data [58]. The Big Data idea, inseparable from the huge increase in data available to various organizations or individuals, creates opportunities for access to valuable analyses, conclusions and enables making more accurate decisions [6, 11, 59].

The Big Data concept is constantly evolving and currently it does not focus on huge amounts of data, but rather on the process of creating value from this data [52]. Big Data is collected from various sources that have different data properties and are processed by different organizational units, resulting in creation of a Big Data chain [36]. The aim of the organizations is to manage, process and analyze Big Data. In the healthcare sector, Big Data streams consist of various types of data, namely [8, 51]:

- clinical data, i.e. data obtained from electronic medical records, data from hospital information systems, image centers, laboratories, pharmacies and other organizations providing health services, patient generated health data, physician’s free-text notes, genomic data, physiological monitoring data [4],
- biometric data provided from various types of devices that monitor weight, pressure, glucose level, etc.,
- financial data, constituting a full record of economic operations reflecting the conducted activity,
- data from scientific research activities, i.e. results of research, including drug research, design of medical devices and new methods of treatment,
- data provided by patients, including description of preferences, level of satisfaction, information from systems for self-monitoring of their activity: exercises, sleep, meals consumed, etc.
- data from social media.

These data are provided not only by patients but also by organizations and institutions, as well as by various types of monitoring devices, sensors or instruments [16]. Data that has been generated so far in the healthcare sector is stored in both paper and digital form. Thus, the essence and the specificity of the process of Big Data analyses means that organizations need to face new technological and organizational challenges [67]. The healthcare sector has always generated huge amounts of data and this is connected, among others, with the need to store medical records of patients. However, the problem with Big Data in healthcare is not limited to an overwhelming volume but also an unprecedented diversity in terms of types, data formats and speed with which it should be analyzed in order to provide the necessary information on an ongoing basis [3]. It is also difficult to apply traditional tools and methods for management of unstructured data [67]. Due to the diversity and quantity of data sources that are growing all the time, advanced analytical tools and technologies, as well as Big Data analysis methods which can meet and exceed the possibilities of managing healthcare data, are needed [3, 68].

**Fig. 1:**
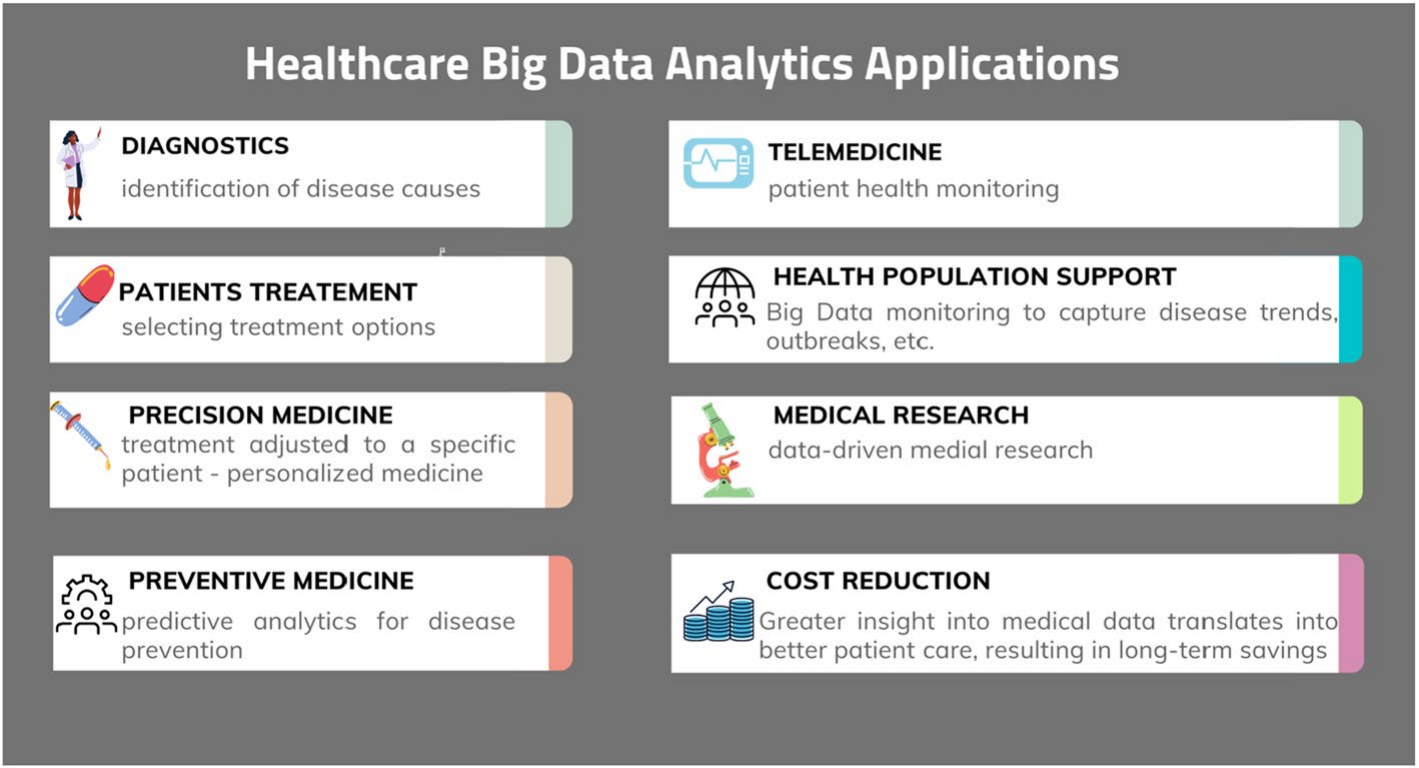
Healthcare Big Data Analytics applications (Source: Own elaboration)

Therefore, the potential is seen in Big Data analyses, especially in the aspect of improving the quality of medical care, saving lives or reducing costs [30]. Extracting from this tangle of given association rules, patterns and trends will allow health service providers and other stakeholders in the healthcare sector to offer more accurate and more insightful diagnoses of patients, personalized treatment, monitoring of the patients, preventive medicine, support of medical research and health population, as well as better quality of medical services and patient care while, at the same time, the ability to reduce costs (Fig. 1).

**Fig. 2:**
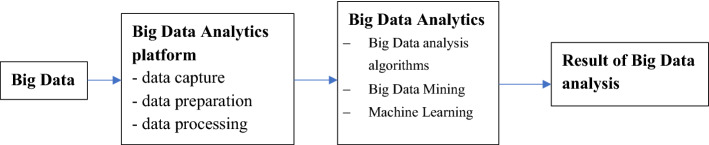
Process of Big Data Analytics (Source: Own elaboration)

The main challenge with Big Data is how to handle such a large amount of information and use it to make data-driven decisions in plenty of areas [64]. In the context of healthcare data, another major challenge is to adjust big data storage, analysis, presentation of analysis results and inference basing on them in a clinical setting. Data analytics systems implemented in healthcare are designed to describe, integrate and present complex data in an appropriate way so that it can be understood better (Fig. 2). This would improve the efficiency of acquiring, storing, analyzing and visualizing big data from healthcare [71].

The result of data processing with the use of Big Data Analytics is appropriate data storytelling which may contribute to making decisions with both lower risk and data support. This, in turn, can benefit healthcare stakeholders. To take advantage of the potential massive amounts of data in healthcare and to ensure that the right intervention to the right patient is properly timed, personalized, and potentially beneficial to all components of the healthcare system such as the payer, patient, and management, analytics of large datasets must connect communities involved in data analytics and healthcare informatics [49]. Big Data Analytics can provide insight into clinical data and thus facilitate informed decision-making about the diagnosis and treatment of patients, prevention of diseases or others. Big Data Analytics can also improve the efficiency of healthcare organizations by realizing the data potential [3, 62].

Big Data Analytics in medicine and healthcare refers to the integration and analysis of a large amount of complex heterogeneous data, such as various omics (genomics, epigenomics, transcriptomics, proteomics, metabolomics, interactomics, pharmacogenetics, deasomics), biomedical data, talemedicine data (sensors, medical equipment data) and electronic health records data [46, 65].

When analyzing the phenomenon of Big Data in the healthcare sector, it should be noted that it can be considered from the point of view of three areas: epidemiological, clinical and business.

From a clinical point of view, the Big Data analysis aims to improve the health and condition of patients, enable long-term predictions about their health status and implementation of appropriate therapeutic procedures. Ultimately, the use of data analysis in medicine is to allow the adaptation of therapy to a specific patient, that is personalized medicine (precision, personalized medicine).

From an epidemiological point of view, it is desirable to obtain an accurate prognosis of morbidity in order to implement preventive programs in advance.

In the business context, Big Data analysis may enable offering personalized packages of commercial services or determining the probability of individual disease and infection occurrence. It is worth noting that Big Data means not only the collection and processing of data but, most of all, the inference and visualization of data necessary to obtain specific business benefits.

In order to introduce new management methods and new solutions in terms of effectiveness and transparency, it becomes necessary to make data more accessible, digital, searchable, as well as analyzed and visualized.

Erickson and Rothberg state that the information and data do not reveal their full value until insights are drawn from them. Data becomes useful when it enhances decision making and decision making is enhanced only when analytical techniques are used and an element of human interaction is applied [22].

Thus, healthcare has experienced much progress in usage and analysis of data. A large-scale digitalization and transparency in this sector is a key statement of almost all countries governments policies. For centuries, the treatment of patients was based on the judgment of doctors who made treatment decisions. In recent years, however, Evidence-Based Medicine has become more and more important as a result of it being related to the systematic analysis of clinical data and decision-making treatment based on the best available information [42]. In the healthcare sector, Big Data Analytics is expected to improve the quality of life and reduce operational costs [72, 82]. Big Data Analytics enables organizations to improve and increase their understanding of the information contained in data. It also helps identify data that provides insightful insights for current as well as future decisions [28].

Big Data Analytics refers to technologies that are grounded mostly in data mining: text mining, web mining, process mining, audio and video analytics, statistical analysis, network analytics, social media analytics and web analytics [16, 25, 31]. Different data mining techniques can be applied on heterogeneous healthcare data sets, such as: anomaly detection, clustering, classification, association rules as well as summarization and visualization of those Big Data sets [65]. Modern data analytics techniques explore and leverage unique data characteristics even from high-speed data streams and sensor data [15, 16, 31, 55]. Big Data can be used, for example, for better diagnosis in the context of comprehensive patient data, disease prevention and telemedicine (in particular when using real-time alerts for immediate care), monitoring patients at home, preventing unnecessary hospital visits, integrating medical imaging for a wider diagnosis, creating predictive analytics, reducing fraud and improving data security, better strategic planning and increasing patients’ involvement in their own health.

Big Data Analytics in healthcare can be divided into [33, 73, 74]:

- descriptive analytics in healthcare is used to understand past and current healthcare decisions, converting data into useful information for understanding and analyzing healthcare decisions, outcomes and quality, as well as making informed decisions [33]. It can be used to create reports (i.e. about patients’ hospitalizations, physicians’ performance, utilization management), visualization, customized reports, drill down tables, or running queries on the basis of historical data.
- predictive analytics operates on past performance in an effort to predict the future by examining historical or summarized health data, detecting patterns of relationships in these data, and then extrapolating these relationships to forecast. It can be used to i.e. predict the response of different patient groups to different drugs (dosages) or reactions (clinical trials), anticipate risk and find relationships in health data and detect hidden patterns [62]. In this way, it is possible to predict the epidemic spread, anticipate service contracts and plan healthcare resources. Predictive analytics is used in proper diagnosis and for appropriate treatments to be given to patients suffering from certain diseases [39].
- prescriptive analytics—occurs when health problems involve too many choices or alternatives. It uses health and medical knowledge in addition to data or information. Prescriptive analytics is used in many areas of healthcare, including drug prescriptions and treatment alternatives. Personalized medicine and evidence-based medicine are both supported by prescriptive analytics.
- discovery analytics—utilizes knowledge about knowledge to discover new “inventions” like drugs (drug discovery), previously unknown diseases and medical conditions, alternative treatments, etc.

Although the models and tools used in descriptive, predictive, prescriptive, and discovery analytics are different, many applications involve all four of them [62]. Big Data Analytics in healthcare can help enable personalized medicine by identifying optimal patient-specific treatments. This can influence the improvement of life standards, reduce waste of healthcare resources and save costs of healthcare [56, 63, 71]. The introduction of large data analysis gives new analytical possibilities in terms of scope, flexibility and visualization. Techniques such as data mining (computational pattern discovery process in large data sets) facilitate inductive reasoning and analysis of exploratory data, enabling scientists to identify data patterns that are independent of specific hypotheses. As a result, predictive analysis and real-time analysis becomes possible, making it easier for medical staff to start early treatments and reduce potential morbidity and mortality. In addition, document analysis, statistical modeling, discovering patterns and topics in document collections and data in the EHR, as well as an inductive approach can help identify and discover relationships between health phenomena.

Advanced analytical techniques can be used for a large amount of existing (but not yet analytical) data on patient health and related medical data to achieve a better understanding of the information and results obtained, as well as to design optimal clinical pathways [62]. Big Data Analytics in healthcare integrates analysis of several scientific areas such as bioinformatics, medical imaging, sensor informatics, medical informatics and health informatics [65]. Big Data Analytics in healthcare allows to analyze large datasets from thousands of patients, identifying clusters and correlation between datasets, as well as developing predictive models using data mining techniques [65]. Discussing all the techniques used for Big Data Analytics goes beyond the scope of a single article [25].

The success of Big Data analysis and its accuracy depend heavily on the tools and techniques used to analyze the ability to provide reliable, up-to-date and meaningful information to various stakeholders [12]. It is believed that the implementation of big data analytics by healthcare organizations could bring many benefits in the upcoming years, including lowering health care costs, better diagnosis and prediction of diseases and their spread, improving patient care and developing protocols to prevent re-hospitalization, optimizing staff, optimizing equipment, forecasting the need for hospital beds, operating rooms, treatments, and improving the drug supply chain [71].

## Challenges and potential benefits of using Big Data Analytics in healthcare

Modern analytics gives possibilities not only to have insight in historical data, but also to have information necessary to generate insight into what may happen in the future. Even when it comes to prediction of evidence-based actions. The emphasis on reform has prompted payers and suppliers to pursue data analysis to reduce risk, detect fraud, improve efficiency and save lives. Everyone—payers, providers, even patients—are focusing on doing more with fewer resources. Thus, some areas in which enhanced data and analytics can yield the greatest results include various healthcare stakeholders (Table 1).

**Table 1:** The use of analytics by various healthcare stakeholders Source: own elaboration on the basis of [19, 20]

The use of analytics by healthcare providers	Healthcare providers are the main recipients and users of analytical systems in healthcare. Thanks to the introduction of electronic medical records, medical facilities will have access to data and the possibility of using analytical systems, enabling the compilation of health services, maximizing its usefulness, profitability, taking into account market demand, costs and without reducing the quality of services. They will be able to securely share patient data between themselves and other entities providing health services. The use of analytics will allow access to statistical forecasts and it will allow to estimate the likelihood of occurrence of specific diseases and, on this basis, to plan types of health services. Thanks to analytics, medical centers will have a complete picture of their activities, taking into account the clinical, management, financial and quality perspectives
The use of analytics by the Payer	The analytics will allow payers to develop plans for managing health and preventive programs, so it can be a factor in improving the quality of patients' health insurance and improving the health and quality of life of insured persons. It will be possible to carry out analyses allowing to determine the structure and cost-effectiveness of medical procedures for a given disease or the risk of its occurrence. Access to cross-sectional information about the consumers will enable payers to identify factors (genetic, demographic or environmental) affecting the emergence and development of specific diseases. It will allow to plan contracting services and implement information and preventive programs, as well as informing patients what diseases they might come across or what are the risks
Analytics in the field of Life Sciences	In pharmaceutical forms and companies producing medical equipment, analytics has been used for several years, as these industries evolve very quickly. Current analytical systems are slowly adapting to the challenges of personalized medicine, allowing the adaptation of treatments, prophylaxis to individual patient genomes, their proteomes and metabolic attributes. Effective solutions in this area have not yet been fully developed. Pharmaceutical companies also use drug sales data to plan marketing activities to achieve greater sales efficiency
The use of analytics by patients	Patients are the final recipients of healthcare, so they will also have to become. Analytics may be useful for finding the best medical facilities and doctors, checking the effectiveness of treatments and medicines ordered, as well as comparing the price and quality of offers of different providers and selecting the best one. The analytical capabilities in the patient area are of course related to the introduction of the Health 2.0 concept thanks to which patients have access to health information from the level of a web browser and can use analytical systems in the same way. Analytical reports will have to be simplified so that patients can understand them

Healthcare organizations see the opportunity to grow through investments in Big Data Analytics. In recent years, by collecting medical data of patients, converting them into Big Data and applying appropriate algorithms, reliable information has been generated that helps patients, physicians and stakeholders in the health sector to identify values and opportunities [31]. It is worth noting that there are many changes and challenges in the structure of the healthcare sector. Digitization and effective use of Big Data in healthcare can bring benefits to every stakeholder in this sector. A single doctor would benefit the same as the entire healthcare system. Potential opportunities to achieve benefits and effects from Big Data in healthcare can be divided into four groups [8]:

1. Improving the quality of healthcare services:
  assessment of diagnoses made by doctors and the manner of treatment of diseases indicated by them based on the decision support system working on Big Data collections,
  detection of more effective, from a medical point of view, and more cost-effective ways to diagnose and treat patients,
  analysis of large volumes of data to reach practical information useful for identifying needs, introducing new health services, preventing and overcoming crises,
  prediction of the incidence of diseases,
  detecting trends that lead to an improvement in health and lifestyle of the society,
  analysis of the human genome for the introduction of personalized treatment.
2. Supporting the work of medical personnel
  doctors’ comparison of current medical cases to cases from the past for better diagnosis and treatment adjustment,
  detection of diseases at earlier stages when they can be more easily and quickly cured,
  detecting epidemiological risks and improving control of pathogenic spots and reaction rates,
  identification of patients who are predicted to have the highest risk of specific, life-threatening diseases by collating data on the history of the most common diseases, in healing people with reports entering insurance companies,
  health management of each patient individually (personalized medicine) and health management of the whole society,
  capturing and analyzing large amounts of data from hospitals and homes in real time, life monitoring devices to monitor safety and predict adverse events,
  analysis of patient profiles to identify people for whom prevention should be applied, lifestyle change or preventive care approach,
  the ability to predict the occurrence of specific diseases or worsening of patients’ results,
  predicting disease progression and its determinants, estimating the risk of complications,
  detecting drug interactions and their side effects.
3. Supporting scientific and research activity
  supporting work on new drugs and clinical trials thanks to the possibility of analyzing “all data” instead of selecting a test sample,
  the ability to identify patients with specific, biological features that will take part in specialized clinical trials,
  selecting a group of patients for which the tested drug is likely to have the desired effect and no side effects,
  using modeling and predictive analysis to design better drugs and devices.
4. Business and management
  reduction of costs and counteracting abuse and counseling practices,
  faster and more effective identification of incorrect or unauthorized financial operations in order to prevent abuse and eliminate errors,
  increase in profitability by detecting patients generating high costs or identifying doctors whose work, procedures and treatment methods cost the most and offering them solutions that reduce the amount of money spent,
  identification of unnecessary medical activities and procedures, e.g. duplicate tests.

According to research conducted by Wang, Kung and Byrd, Big Data Analytics benefits can be classified into five categories: IT infrastructure benefits (reducing system redundancy, avoiding unnecessary IT costs, transferring data quickly among healthcare IT systems, better use of healthcare systems, processing standardization among various healthcare IT systems, reducing IT maintenance costs regarding data storage), operational benefits (improving the quality and accuracy of clinical decisions, processing a large number of health records in seconds, reducing the time of patient travel, immediate access to clinical data to analyze, shortening the time of diagnostic test, reductions in surgery-related hospitalizations, exploring inconceivable new research avenues), organizational benefits (detecting interoperability problems much more quickly than traditional manual methods, improving cross-functional communication and collaboration among administrative staffs, researchers, clinicians and IT staffs, enabling data sharing with other institutions and adding new services, content sources and research partners), managerial benefits (gaining quick insights about changing healthcare trends in the market, providing members of the board and heads of department with sound decision-support information on the daily clinical setting, optimizing business growth-related decisions) and strategic benefits (providing a big picture view of treatment delivery for meeting future need, creating high competitive healthcare services) [73].

The above specification does not constitute a full list of potential areas of use of Big Data Analysis in healthcare because the possibilities of using analysis are practically unlimited. In addition, advanced analytical tools allow to analyze data from all possible sources and conduct cross-analyses to provide better data insights [26]. For example, a cross-analysis can refer to a combination of patient characteristics, as well as costs and care results that can help identify the best, in medical terms, and the most cost-effective treatment or treatments and this may allow a better adjustment of the service provider’s offer [62].

In turn, the analysis of patient profiles (e.g. segmentation and predictive modeling) allows identification of people who should be subject to prophylaxis, prevention or should change their lifestyle [8]. Shortened list of benefits for Big Data Analytics in healthcare is presented in paper [3] and consists of: better performance, day-to-day guides, detection of diseases in early stages, making predictive analytics, cost effectiveness, Evidence Based Medicine and effectiveness in patient treatment.

Summarizing, healthcare big data represents a huge potential for the transformation of healthcare: improvement of patients’ results, prediction of outbreaks of epidemics, valuable insights, avoidance of preventable diseases, reduction of the cost of healthcare delivery and improvement of the quality of life in general [1]. Big Data also generates many challenges such as difficulties in data capture, data storage, data analysis and data visualization [15]. The main challenges are connected with the issues of: data structure (Big Data should be user-friendly, transparent, and menu-driven but it is fragmented, dispersed, rarely standardized and difficult to aggregate and analyze), security (data security, privacy and sensitivity of healthcare data, there are significant concerns related to confidentiality), data standardization (data is stored in formats that are not compatible with all applications and technologies), storage and transfers (especially costs associated with securing, storing, and transferring unstructured data), managerial skills, such as data governance, lack of appropriate analytical skills and problems with Real-Time Analytics (health care is to be able to utilize Big Data in real time) [4, 34, 41].

## Methods

The research is based on a critical analysis of the literature, as well as the presentation of selected results of direct research on the use of Big Data Analytics in medical facilities in Poland.

Presented research results are part of a larger questionnaire form on Big Data Analytics. The direct research was based on an interview questionnaire which contained 100 questions with 5-point Likert scale (1—strongly disagree, 2—I rather disagree, 3—I do not agree, nor disagree, 4—I rather agree, 5—I definitely agree) and 4 metrics questions. The study was conducted in December 2018 on a sample of 217 medical facilities (110 private, 107 public). The research was conducted by a specialized market research agency: Center for Research and Expertise of the University of Economics in Katowice.

When it comes to direct research, the selected entities included entities financed from public sources—the National Health Fund (23.5%), and entities operating commercially (11.5%). In the surveyed group of entities, more than a half (64.9%) are hybrid financed, both from public and commercial sources. The diversity of the research sample also applies to the size of the entities, defined by the number of employees. Taking into account proportions of the surveyed entities, it should be noted that in the sector structure, medium-sized (10–50 employees—34% of the sample) and large (51–250 employees—27%) entities dominate. The research was of all-Poland nature, and the entities included in the research sample come from all of the voivodships. The largest group were entities from Łódzkie (32%), Śląskie (18%) and Mazowieckie (18%) voivodships, as these voivodships have the largest number of medical institutions. Other regions of the country were represented by single units. The selection of the research sample was random—layered. As part of medical facilities database, groups of private and public medical facilities have been identified and the ones to which the questionnaire was targeted were drawn from each of these groups. The analyses were performed using the GNU PSPP 0.10.2 software.

The aim of the study was to determine whether medical facilities in Poland use Big Data Analytics and if so, in which areas. Characteristics of the research sample is presented in Table 2.

**Table 2:** Characteristics of the research sample

The form of ownership	
Public	49.31%
Private	50.69%
Type of services provided (due to reimbursement)	
Under a contract with the NFZ (payer)	23.51%
Commercial only	11.52%
Both forms	64.97%
Number of employees	
Up to 9	17.51%
10–50	34.10%
51–250	27.19%
251–500	13.36%
501–1000	5.53%
Over 1000	2.30%
Voivodeship	
Dolnośląskie	3.2%
Kujawsko pomorskie	0.9%
Lubelskie	9.2%
Lubuskie	2.8%
Łódzkie	32.3%
Małopolskie	4.1%
Mazowieckie	18.4%
Opolskie	1.4%
Podkarpackie	1.4%
Podlaskie	1.4%
Pomorskie	1.4%
Śląskie	18.4%
Świętokrzyskie	1.4%
Warmińsko pomorskie	1.4%
Wielkopolskie	0.9%
Zachodniopomorskie	1.4%

The research is non-exhaustive due to the incomplete and uneven regional distribution of the samples, overrepresented in three voivodeships (Łódzkie, Mazowieckie and Śląskie). The size of the research sample (217 entities) allows the authors of the paper to formulate specific conclusions on the use of Big Data in the process of its management.

For the purpose of this paper, the following research hypotheses were formulated: (1) medical facilities in Poland are working on both structured and unstructured data (2) medical facilities in Poland are moving towards data-based healthcare and its benefits.

The paper poses the following research questions and statements that coincide with the selected questions from the research questionnaire:

- From what sources do medical facilities obtain data? What types of data are used by the particular organization, whether structured or unstructured, and to what extent?
- From what sources do medical facilities obtain data?
- In which area organizations are using data and analytical systems (clinical or business)?
- Is data analytics performed based on historical data or are predictive analyses also performed?
- Determining whether administrative and medical staff receive complete, accurate and reliable data in a timely manner?
- Determining whether real-time analyses are performed to support the particular organization’s activities.

## Results and discussion

On the basis of the literature analysis and research study, a set of questions and statements related to the researched area was formulated. The results from the surveys show that medical facilities use a variety of data sources in their operations. These sources are both structured and unstructured data (Table 3).

**Table 3:** Type of data sources used in medical facility (%)

Statement	1	2	3	4	5
Our organization collects and uses structured data (e.g. databases and data warehouses, reports to external entities)	6.17	7.93	23.35	47.58	10.57
Our organization collects and uses unstructured data (Big Data)	13.66	17.18	27.31	28.19	9.25

1—strongly disagree, 2—I disagree, 3—I agree or disagree, 4—I rather agree, 5—I strongly agree

According to the data provided by the respondents, considering the first statement made in the questionnaire, almost half of the medical institutions (47.58%) agreed that they rather collect and use structured data (e.g. databases and data warehouses, reports to external entities) and 10.57% entirely agree with this statement. As much as 23.35% of representatives of medical institutions stated “I agree or disagree”. Other medical facilities do not collect and use structured data (7.93%) and 6.17% strongly disagree with the first statement. Also, the median calculated based on the obtained results (median: 4), proves that medical facilities in Poland collect and use structured data (Table 4).

**Table 4:** Collection and use of data determined by the size of medical facility (number of employees)

Statement	Number of employees	
	Kendall’s Tau (τ)	p
Our organization collects and uses structured data (e.g. databases and data warehouses, reports to external entities)	0.16	< 0.001
Our organization collects and uses unstructured data (Big Data)	0.23	< 0.001

In turn, 28.19% of the medical institutions agreed that they rather collect and use unstructured data and as much as 9.25% entirely agree with this statement. The number of representatives of medical institutions that stated “I agree or disagree” was 27.31%. Other medical facilities do not collect and use structured data (17.18%) and 13.66% strongly disagree with the first statement. In the case of unstructured data the median is 3, which means that the collection and use of this type of data by medical facilities in Poland is lower.

In the further part of the analysis, it was checked whether the size of the medical facility and form of ownership have an impact on whether it analyzes unstructured data (Tables 4 and 5). In order to find this out, correlation coefficients were calculated.

**Table 5:** Collection and use of data determined by the form of ownership of medical facility

	The form of ownership	N	Average	SD	Median	Mann–Whitney U test	p
Our organization collects and uses structured data (e.g. databases and data warehouses, reports to external entities)	Public	107	3.56	0.92	4.00	0.426	0.670
	Private	110	3.45	1.10	4.00		
	All	217	3.51	1.01	4.00		
Our organization collects and uses unstructured data (Big Data)	Public	107	3.15	1.05	3.00	1.330	0.184
	Private	110	2.90	1.32	3.00		
	All	217	3.02	1.20	3.00		

Based on the calculations, it can be concluded that there is a small statistically monotonic correlation between the size of the medical facility and its collection and use of structured data (p < 0.001; τ = 0.16). This means that the use of structured data is slightly increasing in larger medical facilities. The size of the medical facility is more important according to use of unstructured data (p < 0.001; τ = 0.23) (Table 4.).

To determine whether the form of medical facility ownership affects data collection, the Mann–Whitney U test was used. The calculations show that the form of ownership does not affect what data the organization collects and uses (Table 5).

Detailed information on the sources of from which medical facilities collect and use data is presented in the Table 6.

**Table 6:** Data sources used in medical facility

Type of data	1	2	3	4	5	Average	Median
Information publicly available in databases	2.64	3.52	19.38	46.70	23.35	3.88	4
Reports to external units	5.73	7.05	23.35	38.33	21.15	3.65	4
Logs	11.45	21.59	20.26	26.87	15.42	3.14	3
Transaction data	11.01	14.10	20.70	31.72	18.06	3.33	4
E-mails	8.37	10.57	28.19	30.84	17.62	3.41	4
Data from medical devices	11.01	11.45	21.59	29.07	22.47	3.42	4
Data from sensors	15.42	12.78	22.47	27.31	17.62	3.20	3
Social media	21.59	22.03	27.75	16.74	7.49	2.65	3
RFID codes	25.11	17.62	23.79	23.35	5.73	2.65	3
Geolocation data	30.40	19.38	23.35	19.82	2.64	2.42	2
Phone calls	11.45	14.54	21.59	28.63	19.38	3.31	4
Audio and video data	20.26	19.38	19.82	28.63	7.49	2.83	3

1—we do not use at all, 5—we use extensively

The questionnaire results show that medical facilities are especially using information published in databases, reports to external units and transaction data, but they also use unstructured data from e-mails, medical devices, sensors, phone calls, audio and video data (Table 6). Data from social media, RFID and geolocation data are used to a small extent. Similar findings are concluded in the literature studies.

From the analysis of the answers given by the respondents, more than half of the medical facilities have integrated hospital system (HIS) implemented. As much as 43.61% use integrated hospital system and 16.30% use it extensively (Table 7). 19.38% of exanimated medical facilities do not use it at all. Moreover, most of the examined medical facilities (34.80% use it, 32.16% use extensively) conduct medical documentation in an electronic form, which gives an opportunity to use data analytics. Only 4.85% of medical facilities don’t use it at all.

**Table 7:** The use of HIS and electronic documentation in medical facilities (%)

Statement	1	2	3	4	5
1. Integrated hospital system	19.38	3.08	13.22	43.61	16.30
2. Electronic documentation of patients	4.85	3.96	19.82	34.80	32.16

1—we do not use at all, 5—we use extensively

Other problems that needed to be investigated were: whether medical facilities in Poland use data analytics? If so, in what form and in what areas? (Table 8). The analysis of answers given by the respondents about the potential of data analytics in medical facilities shows that a similar number of medical facilities use data analytics in administration and business (31.72% agreed with the statement no. 5 and 12.33% strongly agreed) as in the clinical area (33.04% agreed with the statement no. 6 and 12.33% strongly agreed). When considering decision-making issues, 35.24% agree with the statement "the organization uses data and analytical systems to support business decisions” and 8.37% of respondents strongly agree. Almost 40.09% agree with the statement that “the organization uses data and analytical systems to support clinical decisions (in the field of diagnostics and therapy)” and 15.42% of respondents strongly agree. Exanimated medical facilities use in their activity analytics based both on historical data (33.48% agree with statement 7 and 12.78% strongly agree) and predictive analytics (33.04% agrees with the statement number 8 and 15.86% strongly agree). Detailed results are presented in Table 8.

**Table 8:** Conditions of using Big Data Analytics in medical facilities (%)

Statement	1	2	3	4	5	Average	Median
3. The organization uses data and analytical systems to support clinical decisions (in the field of diagnostics and therapy)	7.49	13.22	19.38	40.09	15.42	3.45	4.00
4. The organization uses data and analytical systems to support business decisions	7.93	15.42	28.63	35.24	8.37	3.22	3.00
5. To support the organization’s activity, the analyst in the area of administration and business is used	9.25	13.66	28.63	31.72	12.33	3.25	3.00
6. To support the organization’s activity, analytics in the clinical area is primarily used	10.13	8.81	31.28	33.04	12.33	3.30	3.00
7. To support the organization’s activity, analyses are made based on historical data	12.33	12.78	24.23	33.48	12.78	3.23	3.00
8. To support the organization’s activity, predictive analyses (forecasts) are performed	10.13	15.42	21.15	33.04	15.86	3.30	4.00
9. Administrative and medical staff receive complete, accurate and reliable data in a timely manner	2.20	10.13	27.75	36.12	19.38	3.63	4.00
10. We conduct analytical planning processes systematically and analyze new opportunities for strategic use of analytics in the area of business and clinical activities	3.96	14.10	28.63	38.33	10.57	3.39	4.00
11. Real-time analyses are performed to support the organization’s activities	11.89	14.98	26.43	28.19	14.10	3.18	3.00

1—strongly disagree, 2—I disagree, 3—I agree or disagree, 4—I rather agree, 5—I strongly agree

Medical facilities focus on development in the field of data processing, as they confirm that they conduct analytical planning processes systematically and analyze new opportunities for strategic use of analytics in business and clinical activities (38.33% rather agree and 10.57% strongly agree with this statement). The situation is different with real-time data analysis, here, the situation is not so optimistic. Only 28.19% rather agree and 14.10% strongly agree with the statement that real-time analyses are performed to support an organization’s activities.

When considering whether a facility’s performance in the clinical area depends on the form of ownership, it can be concluded that taking the average and the Mann–Whitney U test depends. A higher degree of use of analyses in the clinical area can be observed in public institutions.

Whether a medical facility performs a descriptive or predictive analysis do not depend on the form of ownership (p > 0.05). It can be concluded that when analyzing the mean and median, they are higher in public facilities, than in private ones. What is more, the Mann–Whitney U test shows that these variables are dependent from each other (p < 0.05) (Table 9).

**Table 9:** Conditions of using Big Data Analytics in medical facilities determined by the form of ownership of medical facility

	The form of ownership	N	Average	SD	Median	Mann–Whitney U test	p
The organization uses data and analytical systems to support clinical decisions (in the field of diagnostics and therapy)	Public	107	3.64	1.03	4.00	1.987	0.047
	Private	110	3.26	1.23	4.00		
	All	217	3.45	1.15	4.00		
In order to support the organization’s activity, analytics in the clinical area is primarily used	Public	107	3.47	1.03	4.00	2.077	0.038
	Private	110	3.14	1.22	3.00		
	All	217	3.30	1.14	3.00		
In order to support the organization’s activity, analyses are made based on historical data	Public	107	3.35	1.06	4.00	1.200	0.230
	Private	110	3.11	1.35	3.00		
	All	217	3.23	1.22	3.00		
In order to support the organization’s activity, predictive analyses (forecasts) are performed	Public	107	3.34	1.16	4.00	0.217	0.828
	Private	110	3.27	1.30	3.50		
	All	217	3.30	1.23	4.00		

When considering whether a facility’s performance in the clinical area depends on its size, it can be concluded that taking the Kendall’s Tau (τ) it depends (p < 0.001; τ = 0.22), and the correlation is weak but statistically important. This means that the use of data and analytical systems to support clinical decisions (in the field of diagnostics and therapy) increases with the increase of size of the medical facility. A similar relationship, but even less powerful, can be found in the use of descriptive and predictive analyses (Table 10).

**Table 10:** Conditions of using Big Data Analytics in medical facilities determined by the size of medical facility (number of employees)

Statement	Number of employees	
	Kendall’s Tau (τ)	p
The organization uses data and analytical systems to support clinical decisions (in the field of diagnostics and therapy)	0.22	< 0.001
In order to support the organization’s activity, analytics in the clinical area is primarily used	0.27	< 0.001
In order to support the organization’s activity, analyses are made based on historical data	0.17	< 0.001
In order to support the organization’s activity, predictive analyses (forecasts) are performed	0.14	0.002

Considering the results of research in the area of analytical maturity of medical facilities, 8.81% of medical facilities stated that they are at the first level of maturity, i.e. an organization has developed analytical skills and does not perform analyses. As much as 13.66% of medical facilities confirmed that they have poor analytical skills, while 38.33% of the medical facility has located itself at level 3, meaning that “there is a lot to do in analytics”. On the other hand, 28.19% believe that analytical capabilities are well developed and 6.61% stated that analytics are at the highest level and the analytical capabilities are very well developed. Detailed data is presented in Table 11. Average amounts to 3.11 and Median to 3.

**Table 11:** Analytical maturity of examined medical facilities (%)

Level 1. The organization has no developed analytical capabilities and does not perform analyses	8.81
Level 2. Poor analytical capabilities	13.66
Level 3. There is a lot to do in the area of analytics	38.33
Level 4. The analytical capabilities are well developed	28.19
Level 5. The analytical capabilities are very well developed	6.61

The results of the research have enabled the formulation of following conclusions. Medical facilities in Poland are working on both structured and unstructured data. This data comes from databases, transactions, unstructured content of emails and documents, devices and sensors. However, the use of data from social media is smaller. In their activity, they reach for analytics in the administrative and business, as well as in the clinical area. Also, the decisions made are largely data-driven.

In summary, analysis of the literature that the benefits that medical facilities can get using Big Data Analytics in their activities relate primarily to patients, physicians and medical facilities. It can be confirmed that: patients will be better informed, will receive treatments that will work for them, will have prescribed medications that work for them and not be given unnecessary medications [78]. Physician roles will likely change to more of a consultant than decision maker. They will advise, warn, and help individual patients and have more time to form positive and lasting relationships with their patients in order to help people. Medical facilities will see changes as well, for example in fewer unnecessary hospitalizations, resulting initially in less revenue, but after the market adjusts, also the accomplishment [78]. The use of Big Data Analytics can literally revolutionize the way healthcare is practiced for better health and disease reduction.

The analysis of the latest data reveals that data analytics increase the accuracy of diagnoses. Physicians can use predictive algorithms to help them make more accurate diagnoses [45]. Moreover, it could be helpful in preventive medicine and public health because with early intervention, many diseases can be prevented or ameliorated [29]. Predictive analytics also allows to identify risk factors for a given patient, and with this knowledge patients will be able to change their lives what, in turn, may contribute to the fact that population disease patterns may dramatically change, resulting in savings in medical costs. Moreover, personalized medicine is the best solution for an individual patient seeking treatment. It can help doctors decide the exact treatments for those individuals. Better diagnoses and more targeted treatments will naturally lead to increases in good outcomes and fewer resources used, including doctors’ time.

## Conclusion

The quantitative analysis of the research carried out and presented in this article made it possible to determine whether medical facilities in Poland use Big Data Analytics and if so, in which areas. Thanks to the results obtained it was possible to formulate the following conclusions. Medical facilities are working on both structured and unstructured data, which comes from databases, transactions, unstructured content of emails and documents, devices and sensors. According to analytics, they reach for analytics in the administrative and business, as well as in the clinical area. It clearly showed that the decisions made are largely data-driven. The results of the study confirm what has been analyzed in the literature. Medical facilities are moving towards data-based healthcare and its benefits.

In conclusion, Big Data Analytics has the potential for positive impact and global implications in healthcare. Future research on the use of Big Data in medical facilities will concern the definition of strategies adopted by medical facilities to promote and implement such solutions, as well as the benefits they gain from the use of Big Data analysis and how the perspectives in this area are seen.

## Practical implications

This work sought to narrow the gap that exists in analyzing the possibility of using Big Data Analytics in healthcare. Showing how medical facilities in Poland are doing in this respect is an element that is part of global research carried out in this area, including [29, 32, 60].

## Limitations and future directions

The research described in this article does not fully exhaust the questions related to the use of Big Data Analytics in Polish healthcare facilities. Only some of the dimensions characterizing the use of data by medical facilities in Poland have been examined. In order to get the full picture, it would be necessary to examine the results of using structured and unstructured data analytics in healthcare. Future research may examine the benefits that medical institutions achieve as a result of the analysis of structured and unstructured data in the clinical and management areas and what limitations they encounter in these areas. For this purpose, it is planned to conduct in-depth interviews with chosen medical facilities in Poland. These facilities could give additional data for empirical analyses based more on their suggestions. Further research should also include medical institutions from beyond the borders of Poland, enabling international comparative analyses.

Future research in the healthcare field has virtually endless possibilities. These regard the use of Big Data Analytics to diagnose specific conditions [47, 66, 69, 76], propose an approach that can be used in other healthcare applications and create mechanisms to identify “patients like me” [75, 80]. Big Data Analytics could also be used for studies related to the spread of pandemics, the efficacy of covid treatment [18, 79], or psychology and psychiatry studies, e.g. emotion recognition [35].

## Data Availability

The datasets for this study are available on request to the corresponding author.

## References

[1] Abouelmehdi K, Beni-Hessane A, Khaloufi H (2018). Big healthcare data: preserving security and privacy. J Big Data.

[2] Agrawal A, Choudhary A (2019). Health services data: big data analytics for deriving predictive healthcare insights. Health Serv Eval.

[3] Al Mayahi S, Al-Badi A, Tarhini A. Exploring the potential benefits of big data analytics in providing smart healthcare. In: Miraz MH, Excell P, Ware A, Ali M, Soomro S, editors. Emerging technologies in computing—first international conference, iCETiC 2018, proceedings (Lecture Notes of the Institute for Computer Sciences, Social-Informatics and Telecommunications Engineering, LNICST). Cham: Springer; 2018. p. 247–58. 10.1007/978-3-319-95450-9_21.

[4] Bainbridge M, Househ M, Kushniruk A, Borycki E (2019). Big data challenges for clinical and precision medicine. Big data, big challenges: a healthcare perspective: background, issues, solutions and research directions.

[5] Bartuś K, Batko K, Lorek P, Jabłoński M (2017). Business intelligence systems: barriers during implementation. Strategic performance management new concept and contemporary trends.

[6] Bartuś K, Batko K, Lorek P (2017). Diagnoza wykorzystania big data w organizacjach-wybrane wyniki badań. Informatyka Ekonomiczna.

[7] Bartuś K, Batko K, Lorek P (2018). Wykorzystanie rozwiązań business intelligence, competitive intelligence i big data w przedsiębiorstwach województwa śląskiego. Przegląd Organizacji.

[8] Batko K (2016). Możliwości wykorzystania Big Data w ochronie zdrowia. Roczniki Kolegium Analiz Ekonomicznych.

[9] Bi Z, Cochran D (2014). Big data analytics with applications. J Manag Anal.

[10] Boerma T, Requejo J, Victora CG, Amouzou A, Asha G, Agyepong I, Borghi J (2018). Countdown to 2030: tracking progress towards universal coverage for reproductive, maternal, newborn, and child health. Lancet.

[11] Bollier D, Firestone CM (2010). The promise and peril of big data.

[12] Bose R (2008). Competitive intelligence process and tools for intelligence analysis. Ind Manag Data Syst.

[13] Carter P. Big data analytics: future architectures, skills and roadmaps for the CIO: in white paper, IDC sponsored by SAS. 2011. p. 1–16.

[14] Castro EM, Van Regenmortel T, Vanhaecht K, Sermeus W, Van Hecke A (2016). Patient empowerment, patient participation and patient-centeredness in hospital care: a concept analysis based on a literature review. Patient Educ Couns.

[15] Chen H, Chiang RH, Storey VC (2012). Business intelligence and analytics: from big data to big impact. MIS Q.

[16] Chen CP, Zhang CY (2014). Data-intensive applications, challenges, techniques and technologies: a survey on big data. Inf Sci.

[17] Chomiak-Orsa I, Mrozek B (2017). Główne perspektywy wykorzystania big data w mediach społecznościowych. Informatyka Ekonomiczna.

[18] Corsi A, de Souza FF, Pagani RN (2021). Big data analytics as a tool for fighting pandemics: a systematic review of literature. J Ambient Intell Hum Comput.

[19] Davenport TH, Harris JG (2007). Competing on analytics, the new science of winning.

[20] Davenport TH (2014). Big data at work: dispelling the myths, uncovering the opportunities.

[21] De Cnudde S, Martens D (2015). Loyal to your city? A data mining analysis of a public service loyalty program. Decis Support Syst.

[22] Erickson S, Rothberg H, Rodriguez E (2017). Data, information, and intelligence. The analytics process.

[23] Fang H, Zhang Z, Wang CJ, Daneshmand M, Wang C, Wang H (2015). A survey of big data research. IEEE Netw.

[24] Fredriksson C. Organizational knowledge creation with big data. A case study of the concept and practical use of big data in a local government context. 2016. https://www.abo.fi/fakultet/media/22103/fredriksson.pdf.

[25] Gandomi A, Haider M (2015). Beyond the hype: big data concepts, methods, and analytics. Int J Inf Manag.

[26] Groves P, Kayyali B, Knott D, Van Kuiken S. The ‘big data’ revolution in healthcare. Accelerating value and innovation. 2015. http://www.pharmatalents.es/assets/files/Big_Data_Revolution.pdf (Reading: 10.04.2019).

[27] Gupta V, Rathmore N (2013). Deriving business intelligence from unstructured data. Int J Inf Comput Technol.

[28] Gupta V, Singh VK, Ghose U, Mukhija P (2019). A quantitative and text-based characterization of big data research. J Intell Fuzzy Syst.

[29] Hampel HOBS, O’Bryant SE, Castrillo JI, Ritchie C, Rojkova K, Broich K, Escott-Price V (2016). PRECISION MEDICINE-the golden gate for detection, treatment and prevention of Alzheimer’s disease. J Prev Alzheimer’s Dis.

[30] Harerimana GB, Jang J, Kim W, Park HK (2018). Health big data analytics: a technology survey. IEEE Access.

[31] Hu H, Wen Y, Chua TS, Li X (2014). Toward scalable systems for big data analytics: a technology tutorial. IEEE Access.

[32] Hussain S, Hussain M, Afzal M, Hussain J, Bang J, Seung H, Lee S (2019). Semantic preservation of standardized healthcare documents in big data. Int J Med Inform.

[33] Islam MS, Hasan MM, Wang X, Germack H (2018). A systematic review on healthcare analytics: application and theoretical perspective of data mining. Healthcare.

[34] Ismail A, Shehab A, El-Henawy IM (2019). Healthcare analysis in smart big data analytics: reviews, challenges and recommendations. Security in smart cities: models, applications, and challenges.

[35] Jain N, Gupta V, Shubham S (2021). Understanding cartoon emotion using integrated deep neural network on large dataset. Neural Comput Appl.

[36] Janssen M, van der Voort H, Wahyudi A (2017). Factors influencing big data decision-making quality. J Bus Res.

[37] Jordan SR (2014). Beneficence and the expert bureaucracy. Public Integr.

[38] Knapp MM (2013). Big data. J Electron Resourc Med Libr.

[39] Koti MS, Alamma BH (2019). Predictive analytics techniques using big data for healthcare databases. Smart intelligent computing and applications.

[40] Krumholz HM (2014). Big data and new knowledge in medicine: the thinking, training, and tools needed for a learning health system. Health Aff.

[41] Kruse CS, Goswamy R, Raval YJ, Marawi S (2016). Challenges and opportunities of big data in healthcare: a systematic review. JMIR Med Inform.

[42] Kyoungyoung J, Gang HK (2013). Potentiality of big data in the medical sector: focus on how to reshape the healthcare system. Healthc Inform Res.

[43] Laney D. Application delivery strategies 2011. http://blogs.gartner.com/doug-laney/files/2012/01/ad949-3D-Data-Management-Controlling-Data-Volume-Velocity-and-Variety.pdf.

[44] Lee IK, Wang CC, Lin MC, Kung CT, Lan KC, Lee CT (2020). Effective strategies to prevent coronavirus disease-2019 (COVID-19) outbreak in hospital. J Hosp Infect.

[45] Lerner I, Veil R, Nguyen DP, Luu VP, Jantzen R (2018). Revolution in health care: how will data science impact doctor-patient relationships?. Front Public Health.

[46] Lytras MD, Papadopoulou P (2017). Applying big data analytics in bioinformatics and medicine.

[47] Ma K, et al. Big data in multiple sclerosis: development of a web-based longitudinal study viewer in an imaging informatics-based eFolder system for complex data analysis and management. In: Proceedings volume 9418, medical imaging 2015: PACS and imaging informatics: next generation and innovations. 2015. p. 941809. 10.1117/12.2082650.

[48] Mach-Król M. Analiza i strategia big data w organizacjach. In: Studia i Materiały Polskiego Stowarzyszenia Zarządzania Wiedzą. 2015;74:43–55.

[49] Madsen LB (2014). Data-driven healthcare: how analytics and BI are transforming the industry.

[50] Manyika J, Chui M, Brown B, Bughin J, Dobbs R, Roxburgh C, Hung BA (2011). Big data: the next frontier for innovation, competition, and productivity.

[51] Marconi K, Dobra M, Thompson C, Liebowitz J (2012). The use of big data in healthcare. Big data and business analytics.

[52] Mehta N, Pandit A (2018). Concurrence of big data analytics and healthcare: a systematic review. Int J Med Inform.

[53] Michel M, Lupton D (2016). Toward a manifesto for the ‘public understanding of big data’. Public Underst Sci.

[54] Mikalef P, Krogstie J (2018). Big data analytics as an enabler of process innovation capabilities: a configurational approach. International conference on business process management.

[55] Mohammadi M, Al-Fuqaha A, Sorour S, Guizani M (2018). Deep learning for IoT big data and streaming analytics: a survey. IEEE Commun Surv Tutor.

[56] Nambiar R, Bhardwaj R, Sethi A, Vargheese R. A look at challenges and opportunities of big data analytics in healthcare. In: 2013 IEEE international conference on big data; 2013. p. 17–22.

[57] Ohlhorst F (2012). Big data analytics: turning big data into big money.

[58] Olszak C, Mach-Król M (2018). A conceptual framework for assessing an organization’s readiness to adopt big data. Sustainability.

[59] Olszak CM (2016). Toward better understanding and use of business intelligence in organizations. Inf Syst Manag.

[60] Palanisamy V, Thirunavukarasu R (2017). Implications of big data analytics in developing healthcare frameworks—a review. J King Saud Univ Comput Inf Sci.

[61] Provost F, Fawcett T (2013). Data science and its relationship to big data and data-driven decisionmaking. Big Data.

[62] Raghupathi W, Raghupathi V (2013). An overview of health analytics. J Health Med Inform.

[63] Raghupathi W, Raghupathi V (2014). Big data analytics in healthcare: promise and potential. Health Inf Sci Syst.

[64] Ratia M, Myllärniemi J. Beyond IC 4.0: the future potential of BI-tool utilization in the private healthcare, conference: proceedings IFKAD, 2018 at: Delft, The Netherlands.

[65] Ristevski B, Chen M (2018). Big data analytics in medicine and healthcare. J Integr Bioinform.

[66] Rumsfeld JS, Joynt KE, Maddox TM (2016). Big data analytics to improve cardiovascular care: promise and challenges. Nat Rev Cardiol.

[67] Schmarzo B (2013). Big data: understanding how data powers big business.

[68] Senthilkumar SA, Rai BK, Meshram AA, Gunasekaran A, Chandrakumarmangalam S (2018). Big data in healthcare management: a review of literature. Am J Theor Appl Bus.

[69] Shubham S, Jain N, Gupta V (2021). Identify glomeruli in human kidney tissue images using a deep learning approach. Soft Comput.

[70] Thuemmler C, Thuemmler C, Bai C (2017). The case for health 4.0. Health 4.0: how virtualization and big data are revolutionizing healthcare.

[71] Tsai CW, Lai CF, Chao HC (2015). Big data analytics: a survey. J Big Data.

[72] Wamba SF, Gunasekaran A, Akter S, Ji-fan RS, Dubey R, Childe SJ (2017). Big data analytics and firm performance: effects of dynamic capabilities. J Bus Res.

[73] Wang Y, Byrd TA (2017). Business analytics-enabled decision-making effectiveness through knowledge absorptive capacity in health care. J Knowl Manag.

[74] Wang Y, Kung L, Wang W, Yu C, Cegielski CG (2018). An integrated big data analytics-enabled transformation model: application to healthcare. Inf Manag.

[75] Wicks P (2018). Scaling PatientsLikeMe via a “generalized platform” for members with chronic illness: web-based survey study of benefits arising. J Med Internet Res.

[76] Willems SM (2019). The potential use of big data in oncology. Oral Oncol.

[77] Williams N, Ferdinand NP, Croft R (2014). Project management maturity in the age of big data. Int J Manag Proj Bus.

[78] Winters-Miner LA. Seven ways predictive analytics can improve healthcare. Medical predictive analytics have the potential to revolutionize healthcare around the world. 2014. https://www.elsevier.com/connect/seven-ways-predictive-analytics-can-improve-healthcare (Reading: 15.04.2019).

[79] Wu J (2020). Application of big data technology for COVID-19 prevention and control in China: lessons and recommendations. J Med Internet Res.

[80] Yan L, Peng J, Tan Y (2015). Network dynamics: how can we find patients like us?. Inf Syst Res.

[81] Yang JJ, Li J, Mulder J, Wang Y, Chen S, Wu H, Pan H (2015). Emerging information technologies for enhanced healthcare. Comput Ind.

[82] Zhang Q, Yang LT, Chen Z, Li P (2018). A survey on deep learning for big data. Inf Fusion.

